# The Border Health Consortium of the Californias—Forming a Binational (California–Baja California) Entity to Address the Health of a Border Region: A Case Study

**DOI:** 10.3389/fpubh.2017.00368

**Published:** 2018-01-19

**Authors:** Justine Kozo, Rogelio Zapata-Garibay, María Gudelia Rangel-Gomez, April Fernandez, Ricardo Hirata-Okamoto, Wilma Wooten, Adriana Vargas-Ojeda, Barbara Jiménez, Hector Zepeda-Cisneros, Charles Edwards Matthews

**Affiliations:** ^1^County of San Diego, Health and Human Services Agency, San Diego, CA, United States; ^2^US-Mexico Border Health Commission, Tijuana, Mexico; ^3^Office of Binational Border Health, California Department of Public Health, San Diego, CA, United States; ^4^Keisen Consultores, Mexico City, Mexico; ^5^Universidad Autónoma de Baja California, Tijuana, Mexico; ^6^Instituto de Salud Pública del Estado de Baja California, Tijuana, Mexico

**Keywords:** border health, US–Mexico border, binational health, binational collaboration, global health, international collaboration

## Abstract

The California–Baja California border region is one of the most frequently traversed areas in the world with a shared population, environment, and health concerns. The Border Health Consortium of the Californias (the “Consortium”) was formed in 2013 to bring together leadership working in the areas of public health, health care, academia, government, and the non-profit sector, with the goal of aligning efforts to improve health outcomes in the region. The Consortium utilizes a Collective Impact framework which supports a shared vision for a healthy border region, mutually reinforcing activities among member organizations and work groups, and a binational executive committee that ensures continuous communication and progress toward meeting its goals. The Consortium is comprised of four binational work groups which address human immunodeficiency virus, tuberculosis, obesity, and mental health, all mutual priorities in the border region. The Consortium holds two general binational meetings each year alternating between California and Baja California. The work groups meet regularly to share information, resources and provide binational training opportunities. Since inception, the Consortium has been successful in strengthening binational communication, coordination, and collaboration by providing an opportunity for individuals to meet one another, learn about each other systems, and foster meaningful relationships. With binational leadership support and commitment, the Consortium could certainly be replicated in other border jurisdictions both nationally and internationally. The present article describes the background, methodology, accomplishments, challenges, and lessons learned in forming the Consortium.

## Introduction

The California–Baja California border region is characterized as one of the most frequently traversed areas in the world, with over 100,000,000 border crossings per year (1). Many individuals living in the region spend significant time in both countries for various reasons, including employment, recreation, family, and health care (2). From a public health perspective, the cities along the border are considered one region with shared experiences, economic and environmental conditions, as well as health and disease (3).

The region’s main health challenges include cardiovascular and metabolic disease, diabetes mellitus and obesity, chronic viral hepatitis and tuberculosis (TB) (4), human immunodeficiency virus (HIV) and other sexual transmitted diseases (5), breast and cervical cancer, asthma and other respiratory diseases (6), as well as substance abuse (7) and mental health (8). Many of these health challenges are exacerbated by migration and acculturation patterns which cause significant changes in diet, occupation, access to health care, and environmental exposures. For instance, individuals seeking employment in the manufacturing industry may be exposed to lead poisoning and other environmental pollutants which can create new or worsen existing health conditions (8).

There are hundreds of entities (e.g., Federal, State, Municipal, County, Non-profit organizations, Nongovernmental Organizations, Universities, etc.) in the region addressing many binational health areas, such as disease surveillance and prevention, research, emergency preparedness, health-care access, and public health academic partnerships. However, many have traditionally worked independently and often disconnected from others working on the same issues. Where they align is around the shared goals of improving health and safety, increasing regular communication and exploring opportunities for collaboration. Recognition of these shared goals and the mutual interest of creating more connections and opportunities for binational collaboration were the motivation behind creating the Border Health Consortium of the Californias.

## Background and Rationale

In 2011, graduates of the *Leaders Across Borders* binational leadership development program, sponsored and funded by the US–Mexico Border Health Commission (BHC), addressed the need for a structured meeting space to improve binational collaboration (9). The group organized under the name of the *San Diego Border Health Collaborative* (10). Facilitated by the Public Health Services Division of the County of San Diego, Health and Human Services Agency and the California Department of Public Health, Office of Binational Border Health, formal meetings began taking place in San Diego with participation from mostly local universities and non-profit organizations. On February 17, 2011, the group began meeting regularly to share information on local projects within San Diego. From the very beginning, participants expressed the need and urgency to connect with Baja California to establish a binational meeting structure. Prior to 2011, there were other binational coalitions in the region and for various reasons, they were no longer active. Members felt that if they were going to continue meeting around border health issues, the only way to move forward was in collaboration with Baja California.

## Integration Process

In the spring of 2012, a group of San Diego Border Health Collaborative members requested a meeting with the Executive Secretary of the Mexico Section of the US–Mexico BHC, to discuss the possibility of creating a binational border health group. The Executive Secretary supported the idea and offered guidance and input on the structure and formation. The Executive Secretary emphasized the following key points: identify the top health priorities on both sides of the border; ensure equal leadership representation from both countries; develop a process that allows for meaningful engagement, while respecting existing systems within each country; and create bylaws to govern the Consortium’s leadership and process. In the spring of 2013, after approximately 12 months of collaborative planning, the Executive Secretary informed the State of Baja California Secretary of Health of the proposal and solicited his support. The Secretary of Health agreed and officially designated representatives to participate in future meetings.

On November 7, 2013, the first binational meeting was held in Tijuana, Baja California with 43 attendees representing agencies and organizations primarily from Tijuana and San Diego. Among the participants included the Baja California Secretary of Health; representatives from the US and Mexican Consulates; local universities; directors of non-profit agencies; and other governmental agencies at the local, state, and federal levels from both the US and Mexico. The concept of a “Border Health Consortium” was presented and followed by a dialogue on shared goals and next steps. The proposal was well received and the majority of participants demonstrated their support in establishing the *Border Health Consortium of the Californias* (now commonly referred to as the “Border Health Consortium” or the “Consortium”).

At the meeting, a survey was disseminated among the participants and the top three advantages for developing the Border Health Consortium were identified as: (1) an opportunity for networking; (2) an opportunity to participate in binational trainings; and (3) an opportunity to disseminate information on border health priorities. Key themes that emerged in discussion included more collaboration among regional medical schools, educating one another on national health-care systems, and identifying general guidelines for cross-border collaboration. In addition, all participants agreed that it was important to evaluate and measure progress. The attendees also visualized the Border Health Consortium as a conduit for organizations to build relationships with key stakeholders and serve as a key partner for strategic funding opportunities in the border region.

## Building Structure

On May 8, 2014, the second Border Health Consortium of the Californias meeting took place in Tijuana with approximately 60 representatives from both California and Baja California. At this meeting, another survey was disseminated to measure interest among participants in forming specific binational work groups. Participants voted on creating work groups to address the following specific health issues: HIV, TB, obesity, and mental health. In addition, participants expressed an interest in creating a binational directory of participating individuals and organizations and scheduling two general binational meetings per year, alternating between California and Baja California. They also voted on creating a Binational Executive Committee to establish and guide the vision and provide strategic direction.

The executive committee is comprised of 12 individuals representing various institutions in San Diego, California and Tijuana, Baja California. From 2014 to present, members include representatives from governmental agencies [the US–Mexico BHC (California and Baja California outreach offices); the California Department of Public Health, Office of Binational Border Health; the City of Tijuana; the San Diego County Health and Human Services Agency], universities (the Universidad Autónoma de Baja California; Universidad Xochilcalco; University of California, San Diego; San Diego State University), and community-based agencies (Fronteras Unidas Pro Salud, A.C; San Diego County Medical Society; and Project Concern International).

In addition to the executive committee, there are three main coordinating agencies that comprise the “Logistical Support Group”: the US–Mexico BHC (the California and Baja California outreach offices), the California Department of Public Health, Office of Binational Border Health and the County of San Diego Health, and Human Services Agency. The Logistical Support Group facilitates meeting planning, manages the member directory and distribution list, and coordinates all meeting logistics.

The executive committee and logistical support group are essential in terms of operational sustainability. Establishing clear roles and responsibilities ensures institutional commitment and support during leadership transitions. The Consortium structure is unprecedented in the US–Mexico border region and a novel component of this successful binational collaboration.

## Methods

On September 18, 2014, the executive committee participated in a 1-day strategic planning session and developed the goals and objectives for the Border Health Consortium. The group felt strongly about being a dependable and trustworthy binational organization. After robust and thorough discussion, the mission was developed: *to be an organized, consolidated, believable, and trustworthy network internationally known for the improvement of health along the California–Baja California Border Region*. The executive committee also determined the Consortium’s four main goals: (1) identify common challenges; (2) use resources efficiently; (3) optimize articulation through collaboration, coordination, and communication (“the three Cs”); and (4) support training and development. A detailed description of the Strategic plan was developed (11). Bylaws were developed at a later date in order to describe the Consortium’s structure, member expectations, work group guidelines, and other aspects related to infrastructure and sustainability.

The Border Health Consortium of the Californias is successful through a framework called *Collective Impact*, a unifying, group approach utilized to address complex issues (12). The Border Health Consortium’s focus is on how member agencies contribute to improving health in the California–Baja California border region. The five components needed to achieve Collective Impact include a shared vision, mutually reinforcing activities, continuous communication, consistent measurement, and a coordinating or “backbone” organization.

### A Shared Vision

The Border Health Consortium member agencies work on a variety of issues, including, but not limited to infectious disease surveillance, emergency preparedness, HIV prevention, human rights, obesity prevention, cancer treatment, mental health, academia, and research. However, they all embody a shared vision of a “Healthy Border Region” and the desire to improve health outcomes among border communities. This shared vision creates commonality among members and is the foundation for building relationships and trust. In addition, other key documents are utilized to create a strategic and operative vision such as the Consortium’s strategic map and other commonly referenced documents, such as Healthy Border 2020 (13).

### Mutually Reinforcing Activities

Members work toward accomplishing the mission and goals set forth by their individual agencies. The Border Health Consortium provides an opportunity for member agencies to align their goals and explore opportunities for collaboration. At every meeting, the Border Health Consortium strategic plan is reviewed, discussed, referenced and serves as a reminder of the broader goals and objectives of the Border Health Consortium.

The work groups also address different health priorities yet support each other’s goals and objectives and collaborate on efforts. For instance, all work groups recognize that mental health is linked to both chronic and infectious disease and they promote the importance of mental health in all their activities. Further, the TB and HIV work groups recently held a conference in October 2017 in which both topics were simultaneously addressed to underscore their interconnectedness and to explore how they can borrow best practices for prevention, disease management, and binational care coordination.

### Continuous Communication

In an effort to support continuous communication, work groups and the executive committee meet frequently to discuss current activities and pressing needs. The Border Health Consortium of the Californias holds binational meetings two times per year in the spring and fall. These meetings alternate between California and Baja California. Each meeting consists of work group presentations and updates, subject matter expert presentations (e.g., research projects, funding opportunities for binational public health projects), and an opportunity for networking. An important criterion is that all topics must be binational in nature, meaning a connection and relevance to both countries, not one alone.

### Consistent Measurement

As part of the Collective Impact approach, it is understood that ongoing evaluation is critical to determine whether or not the entity is achieving its goals and making an impact on the health of the region. It is also important for participating agencies to agree and align with measurement practices. The Border Health Consortium of the Californias has identified this area as a priority and is currently developing a plan to ensure that outcomes are measured in a systematic way.

### Backbone Organization

The Binational Executive Committee functions as the Backbone Organization. Its role is to reinforce the vision and provide strategic direction, support activities, implement evaluation, and address funding needs. For instance, executive committee agencies take turns providing in-kind support, hosting meetings, and providing funds to support necessary items, such as simultaneous interpretation or website support.

## Accomplishments

### Intangible Integration and Benefits

The value of relationship building cannot be overstated. Members describe numerous connections that have been made as a result of participating in Consortium meetings, which have led to several collaborative efforts, such as coordinating binational trainings and conferences, establishing formal agreements between institutions, and communicating with counterparts regarding binational disease surveillance. The opportunity to connect in person is the cornerstone of international collaboration and lasting relationships. These interactions facilitate consistent communication and support when there are any sensitive or emergency issues of mutual concern.

### Tangible Institutional Integration and Collaboration

As of May 2017, the Border Health Consortium of the Californias has held nine binational meetings since its inception in 2014. The meetings are well attended and depending on the location of the meeting, there tends to be more local participation in the host country. At a recent meeting held on May 18, 2017 in San Diego, 70 individuals attended with 52 from California and 18 from Baja California. The meeting addressed the impact of recent socioeconomic and political changes on health in the California–Baja California Border region.

May’s meeting illustrates a key accomplishment of the Border Health Consortium of the Californias which is the ability to bring together leadership representing non-profit organizations, government entities, universities, and community-based agencies in one space to discuss pressing issues and opportunities for binational collaboration in the California, Baja California border region. The level of leadership in attendance is a testimony to the importance of these meetings and the opportunity to gather around shared priorities.

Other important accomplishments include various binational activities accomplished by the work groups. As described previously, the four work groups address HIV, TB, obesity, and mental health.

#### HIV Work Group

The cities of San Diego and Tijuana both experience challenges in HIV prevention and treatment due to a highly mobile population, individuals engaging in high-risk behaviors, and challenges in accessing care (14). Individuals living with HIV in the border region often access medical care in both countries (15). There is a long history of binational HIV collaboration in the California–Baja California border region and many successful outcomes can be attributed to these partnerships. The HIV work group of the Border Health Consortium of the Californias immediately began organizing and tackling priorities, such as establishing a binational directory of care providers and organizing three symposiums in 2015, 2016, and 2017. The symposiums brought together researchers, advocates, providers, and governmental employees to address important issues, such as research, human rights, and coordinating cross-border care provision. The 2017 symposium addressed both HIV and TB in with the goals of sharing best practices around binational care coordination and building relationships among providers in California and Baja California who often have patients in common.

#### TB Work Group

Similar to HIV, the cities of San Diego and Tijuana face many challenges in terms of TB prevention and treatment continuity due to the dynamic movement within the region. The Mexican state of Baja California, at 2013 had the highest incidence of TB in Mexico: 54.4 cases per 100,000 individuals or nearly triple the national average (16). In 2015, California ranked third among the 50 states in TB rates: 5.5 per 100,000 persons (17). The TB work group was formed by experts on both sides of the border with care continuity as the single most important issue. In addition, the work group is focused on creating a space where organizations, governments, funding agencies, and medical professionals can come together to discuss the issues related to TB in the border region; for example, medications and lab capacity among others.

#### Obesity Work Group

Nearly one-third of the adult populations in the US and Mexico are obese, occupying the number one and number two countries with the highest rates of obesity in the world, excluding countries with small populations (18). From the beginning, there was a strong interest in collaboration on this important topic. The Obesity work group has a membership of several agencies and organizations, including local and state government, clinics, universities, and non-profit organizations. The group organized quickly and began meeting regularly to share information about each other’s programs and resources. Through collaboration with the San Diego County Childhood Obesity Initiative, the Obesity group learned about the *5-2-1-0 campaign*, an evidenced-based nationally recognized obesity reduction social marketing campaign (19). The City of Tijuana’s Public Health Director immediately showed interest in the campaign and expressed his desire to implement the campaign in the city of Tijuana. After several months of planning, coordinating with governmental agencies, translating materials, and seeking approval and support, the campaign was implemented in the fall of 2016. This is the first obesity prevention campaign to be implemented in the sister cities of San Diego and Tijuana. This supports the notion that a shared population deserves shared public health messaging and guidance.

#### Mental Health Work Group

Quantifying the magnitude of mental health illness in the border region remains a challenge. Cognizant of this challenge, it is a priority for both California and Baja California and there is a strong desire to collaborate on efforts to address this issue. While there is a long history of mental health collaboration in the California–Baja California border region, partnerships have traditionally been among specific institutions (such as between governmental agencies) and not on a broad scale with diverse agencies. Due to its varied nature, one of the mental health-work group’s goals is to learn about each other’s systems and services. Several meetings and agency tours have taken place among mental health practitioners and leaders in the California/Baja California border region in the last 2 years since the group formed. On October 13, 2017 the group planned and carried out a binational symposium at the Tijuana Mental Health Hospital with over 90 people in attendance. The objective of the symposium was to discuss the experiences and challenges of migrants who move across the US–Mexico border and how this impacts their mental health. Providers, researchers, and other experts shared data as well as powerful stories unique to their patients. A panel of experts discussed the mental health challenges faced by children who are greatly affected by migration due to an increase in anxiety, depression, and behavioral and family changes. The mental health work group participants represent public health, behavioral health, the Mexican consulate in San Diego, Cancer treatment centers, academia and individuals working in various aspects of chronic disease, and mental health.

## Discussion

### Challenges

As with all collaborative endeavors, there are challenges that exist and opportunities for growth. For example, in terms of operations, the following areas have been the center of multiple executive committee meeting discussions: membership, communications, meeting planning, and sustainability. In order to address these areas, the executive committee created bylaws to establish expectations, roles, and responsibilities. Funding is also a challenge as occasional costs arise primarily related to language interpretation. When interpretation is essential, the executive committee member agencies take turns providing funds to support this cost.

The border crossing dynamic presents participation challenges, including hesitancy among individuals new to crossing the border due to the unfamiliarity of the sister city; the additional time commitment due to travel and border wait times; and competing professional priorities (for example, many participants from Baja California are practicing physicians and attending these meetings is a significant burden on their schedule—yet, in spite of this challenge, they still come). In order to mitigate the border crossing challenge, many members offer assistance to others crossing the border for the first time and meeting locations are held close to the ports of entry. There is always a formal recognition and appreciation at every meeting to those who made the effort to attend the meeting.

### Limitations

The primary limitation of the Consortium is the lack of a comprehensive evaluation to demonstrate outcomes and overall efficacy. The binational executive committee has employed process evaluation to capture development and progress. Work group activities and annual meetings disseminate surveys to measure member engagement, satisfaction, and general feedback. The binational executive committee is exploring other evaluation techniques, such as Social Network Analysis and Collaboration Assessment, to capture key collaborations and formal agreements that have resulted from Consortium activities. Once performed and analyzed, this type of evaluation would be useful in identifying concrete results, strengths, and opportunities for improvement.

### Recommendations and Generalizability

There are many other border regions in the US and globally and presumably with similar challenges. Reiterating the components of Collective Impact, the Border Health Consortium of the Californias is successful due to a shared vision for a healthy border region, mutually reinforcing activities of member organizations and work groups, and an executive committee that ensures continuous communication and progress toward measuring results. The format is unique along the US–Mexico border and feedback from other states suggests an interest in forming like entities in other regions. With the commitment of a few key organizations, other border regions could easily replicate the Consortium and following the Collective Impact framework is a promising strategy. Other recommendations include garnering binational leadership support from the outset, ensuring collaborative, equitable decision-making in every phase of development and implementation, and developing structure to ensure progress and sustainability.

To address funding needs, such as interpretation costs, one recommendation is to prioritize which meetings require interpretation and which do not. For instance, the Consortium aims to provide interpretation at the large annual meetings. Now in its fourth year, it has been successful in securing interpretation funds for every annual meeting thanks to contributions of executive committee member organizations. Further, working in a border region there are many bilingual professionals. When funds are unavailable for interpretation (which is the case for almost all work group and executive committee meetings), there are many bilingual individuals who offer assistance with informal language interpretation.

A final recommendation is to recognize that while a region may share a population and many health concerns, priorities are sometimes different. For example, while one country may be focusing resources on educating the public around vector disease prevention, the other country may perceive TB prevention as an urgent issue that warrants more immediate attention. It is critical to be mindful of these important differences and binational collaboration works best when individuals and agencies remain flexible, understanding, and willing to compromise. The Border Health Consortium has been successful in identifying mutual priorities through surveying its members and agreeing on the top four focus areas for the immediate future: HIV, TB, obesity, and mental health. These priorities may shift in years to come and will be determined through additional surveys and consensus among members.

## Conclusion

The Border Health Consortium has been successful in meeting its goals and objectives described in the strategic planning section of this paper. Through surveying its members, it was able to identify common challenges and determine four priority areas: obesity, TB, HIV, and mental Health. Work groups were formed to address these areas and they have made significant progress in terms of developing structure, holding regular binational meetings, setting their own goals and objectives, and carrying out several binational events. Capacity building, professional trainings, and leadership development have been achieved through work group conferences and trainings. These activities support the Consortium’s main goals which are to strengthen binational communication, coordination, and collaboration among professionals and agencies working in the border region.

The Border Health Consortium of the Californias continues to grow through professional connections and referrals. It provides a unique opportunity for individuals and agencies representing academia, government, non-profits, universities, and clinical care to come together in one space, discuss, and exchange ideas and build relationships, all with the common goal of improving health in the California–Baja California border region. Members of the Border Health Consortium appreciate the opportunity to know one another, especially their counterparts and colleagues in their sister city. Allowing a space for people to get to know one another, learn about each other systems, and foster meaningful relationships is the core of successful binational collaboration. Members recognize that they share a community and a population and it is paramount to work together to improve the health of the region.

## Author Contributions

For this article, all the authors conceptualized and designed the topic described in the paper—the Border Health Consortium of the Californias. JK drafted the article and MR-G, AF, RH-O, WW, AV-O, BJ, RZ-G, HZ-C, and CM provided critical revisions of the article. JK, MR-G, AF, RH-O, WW, AV-O, BJ, RZ-G, HZ-C, and CM all approved the final version to be published.

## Conflict of Interest Statement

The authors do not have any conflict of interests to declare. This article was written in the absence of any commercial relationships that could be construed as a potential conflict of interest.
